# An Operation Reduction Using Fast Computation of an Iteration-Based Simulation Method with Microsimulation-Semi-Symbolic Analysis †

**DOI:** 10.3390/e20010062

**Published:** 2018-01-18

**Authors:** Vladimir Mladenovic, Danijela Milosevic, Miroslav Lutovac, Yigang Cen, Matjaz Debevc

**Affiliations:** 1 Faculty of Technical Sciences Cacak, University of Kragujevac, 32000 Cacak, Serbia; 2 The School of Electrical Engineering and Computer Science of Applied Studies Belgrade, 11000 Belgrade, Serbia; 3 School of Computer and Information Technology, Beijing Jiaotong University, 100044 Beijing, China; 4 The Faculty of Electrical Engineering and Computer Science, University of Maribor, 2000 Maribor, Slovenia

**Keywords:** microsimulation-semi-symbolic analysis, iteration-based simulation method, Kummer’s transformation

## Abstract

This paper presents a method for shortening the computation time and reducing the number of math operations required in complex calculations for the analysis, simulation, and design of processes and systems. The method is suitable for education and engineering applications. The efficacy of the method is illustrated with a case study of a complex wireless communication system. The computer algebra system (CAS) was applied to formulate hypotheses and define the joint probability density function of a certain modulation technique. This innovative method was used to prepare microsimulation-semi-symbolic analyses to fully specify the wireless system. The development of an iteration-based simulation method that provides closed form solutions is presented. Previously, expressions were solved using time-consuming numerical methods. Students can apply this method for performance analysis and to understand data transfer processes. Engineers and researchers may use the method to gain insight into the impact of the parameters necessary to properly transmit and detect information, unlike traditional numerical methods. This research contributes to this field by improving the ability to obtain closed form solutions of the probability density function, outage probability, and considerably improves time efficiency with shortened computation time and reducing the number of calculation operations.

## 1. Introduction

In general, theoretical, experimental, and computational approaches are the basis for the study of observed phenomena. Every scientific and experimental result is expected to be placed into a function for its use, so the commercial use of products and services, and many engineering uses, emanate from a scientific approach that has been translated into an engineering approach.

Emerging developments are posing challenges in information technology [1,2,3] that include searching large databases [1,2], solving complex processes described by mathematical models, analyzing phenomena in communications in the information space, such as the transmission of wireless signals in urban environments [4,5,6], and the continuous high speed delivery of information without stagnation in software engineering [7]. The common feature among all these issues is how to directly obtain results for further processing or exploitation. To address this challenge, complex mathematical tools are used to perform analyses and simulations of the performance of the observed processes and systems. Most often, a classical mathematical analysis does not provide final answers in closed form for complex phenomena, so special functions are used to obtain a solution. When results cannot be obtained with mathematical models, we use numerical methods. Most numerical tools include complex calculations, such as differential and integral equations and algebraic structures, using numerical mathematics algorithms such as Newton-Cotes, Romberg integration, Gauss-Christoffel, trapezium rule, Gauss formulas, etc. [8,9]. Due to this complexity, students may not understand the complete process or system, or cannot perform the method performance analysis to the end within the bounds of the classroom. Engineers and researchers may not have appropriate insight into the impact of the parameters necessary for the effective investigation or design. Additionally, the numerical computation generates a large amount of data, which may sometimes lead to erroneous results [10,11]. These incorrect results may be due to the finite word length in the records, or errors occurring during the shortening of numbers in fractions, for example. These methods do not provide the ability to manipulate with analytic expressions. These issues have been overcome by introducing a new method that treats variables and parameters as symbols. The method is called the iteration-based simulation method (IBSM) [11,12]. In addition to the IBSM that provides symbolic analysis, we allow the analysis to be partially observed through the concept of microsimulation analysis, which additionally enhances the ability to influence the parameters and variables. Also, we created an effective method with fast computation time that, together with the operation reduction, provides accurate results quickly. Computer algebra systems are important tools for these analyses, developments, and research, as they provide a completely new approach for understanding and solving complex cases. In this paper, we present the above methods directly applied to two examples. Both examples require complex analysis and use symbolic closed form expressions, and their numerical analysis is time-consuming.

The paper is organized as follows: in Section 2, the problem statement is described. Section 3 illustrates the complete methodology and procedure for applying the method, as well as examples with results in details. In Section 4, the collected results are discussed in detail.

## 2. Problem Statement

A large number of simulations do not guarantee that tolerances will not be exceeded. This is one of the numerous drawbacks of numerical-based tools. Our study had three goals. The first was to solve any analysis in closed form to allow further simplification and manipulation by using an iteration-based simulation method. The second goal was to develop an algorithm to quickly compute the method. Finally, we wanted to reduce the number of operations in the algorithm prior to its implementation. All phases of development and testing were observed by microsimulation-semi-symbolic analysis.

The IBSM was developed using the computer algebra system (CAS) to simplify complex algebraic expressions that offers an acceptable reduced analytic form for further manipulation or simulation as a closed form solution as previously published [12]. As integrals are present in the majority of the analyses, we approached the analyses with elementary calculating when the integrals are presented using Riemann sum. The method converts low-complexity implementation into a high-complexity structure. This approach allows implementation in the hardware environment.

The CAS performs symbolic mathematical operations and is used in the fields of mathematics and computer science. The CAS is based on algebraic calculations and manipulations performed using the same process as manual derivations. The CAS exclusively includes working with symbols, and numerical calculation is a special case for a CAS. Since symbols are used as variables, CAS deals with symbolic processing. Symbolic processing (SP) involves the development, implementation, and application of algorithms that manipulate and analyze mathematical expressions. CAS provides a deeper understanding and helps students to learn and engineers to simulate and design. The Wolfram language (WL) is the programming language suitable for CAS. WL has the ability to manipulate symbolic expressions using a method similar to traditional manual derivation [13]. The WL is characterized by high-performance computing and the generation of compact and short program codes.

The goal of IBSM is to introduce a new parameter to obtain a closed form expression. Since the iteration is a new parameter, we used a transformation to change the integral into a sum, i.e., a series. For this purpose, we used Riemann sum transformation for the features of the improper integrals. Using this method, we obtained closed form expressions that can be manipulated and simplified, with a short computation time, while reducing the operations. The results were tested and verified. The general form of the Riemann integral transformation into a series is given as follows [14,15]:

(1) $$\int_{a}^{b}f(x)dx=\underset{\left\|\Delta x\right\|\to 0}{\lim}\sum_{i=1}^{n}f(x_{i})\Delta x_{i}$$

**Figure 1:**
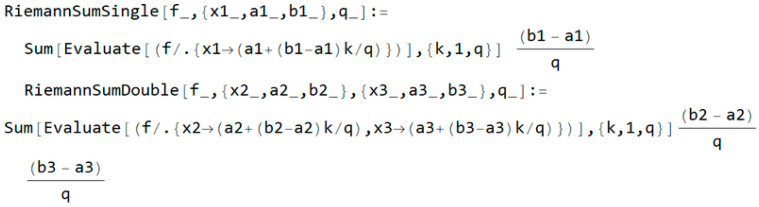
Wolfram language code for a Riemann sum.

By observing the integrals in the previous session, we defined two types of Riemann sums: a single sum and a double sum. We first solved the single sum, then solved the double integrals. So, given Equation (1), the Wolfram language code is shown in Figure 1, where q is the value of the iteration in the defined transformation.

Microsimulation mimics a complex phenomenon by describing its micro-components. Essentially, the system is left free to develop without too many constraints and simplifying assumptions [16]. However, when microsimulation is used with only symbolic content, and the particular numerical values are changed in the final stage, it is called microsimulation semi-symbolic analysis (MSSA). We observed each element of the symbolic calculation through MSSA, which provides faster and better testing and verification as well as a reduction in the operations [17,18]. MSSA also directly calculates in the first run without requiring more simulation attempts.

The next step was the development of an algorithm to allow fast computation. To achieve this, we treated the expression as a series. As a reminder, a short explanation of the concept of fast computation follows. A series is said to converge slowly if a large number of members of the series need to be added to determine a sum with the required accuracy. During the addition of series members using the member-by-member technique, the process automatically occurs and is interrupted when a selected criterion for error evaluation is fulfilled. Given the ultimate summation, the absolute value of the relationship between the last member and the calculated sum is most often used. This criterion is not always reliable, especially for the addition of a trigonometric series. An error caused by an interrupted summing is always higher than estimated. Conversely, contemporary computing machines can quickly add a large number of members in the series. However, due to the limitation on the format of the records in the registers, a certain number of decimal places are eliminated, which leads to the accumulation of errors and to completely absurd results in the process of summing. Therefore, procedures exist for accelerating the convergence of a series, such as Kummer, Aitken, Cesar, and Euler. This paper presents an effective method for accelerating the convergence of a series based on Kummer’s transformation.

We adhered to two theorems. The first states that if $\sum_{k=1}^{n}a_{k}$ convergences, then $\underset{k\to \infty }{\lim}a_{k}=0$. The second states that if $\sum_{k=1}^{n}a_{k}$ and $\sum_{k=1}^{n}b_{k}$ are positive series, and if $\underset{k\to \infty }{\lim}\frac{a_{k}}{b_{k}}=\rho ,\;(b_{k}\neq 0)$, then convergence or divergence occur simultaneously. Kummer’s transformation, better known as Kummer’s acceleration method, accelerates the convergence of many series. The method subtracts from a given convergent series $\sum a_{k}$, and another equivalent series $\sum b_{k}$, whose sum $C=\sum_{k\geq 0}b_{k}$ is well known and finite. Kummer’s transformation is described as:

(2) $$\sum_{k=0}^{\infty }a_{k}=\rho \sum_{k=0}^{\infty }b_{k}+\sum_{k=0}^{\infty }\left(1-\rho \frac{b_{k}}{a_{k}}\right)a_{k}=\rho C+\sum_{k=0}^{\infty }\left(1-\rho \frac{b_{k}}{a_{k}}\right)a_{k}$$

**Figure 2:**
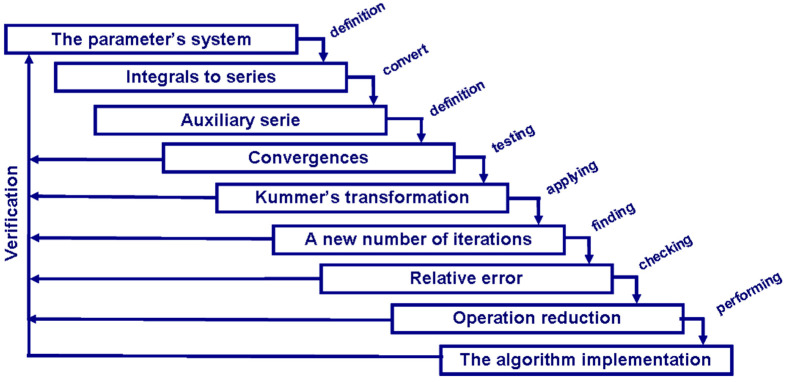
Steps of the speeding up and operation reduction process.

The convergence of the right hand side of Equation (2) is faster because 1 − *ρ*·*b_k_*/*a_k_* tends to 0 as *k* tends to infinity [19]. The complete procedure is shown in Figure 2.

The reduction in operations was performed by counting all math operations and functions contained in the final expressions. Wolfram language allows the performance of direct counting. Mathematical operations and functions in WL can be viewed both symbolically and as commands. Operations are recognized using the FullForm command, and the counting is performed using the StringPosition command. Since we had sums where the numbers are repeated *q* times, the WL code for completely counting the operations is:

InnerOperations=q*FullForm[aK[z,q];]
StringPosition[InnerOperations,{"Times","Power","Plus","Rational", "BesselI","Log","Exp"}];


The orders of Times, Plus, BesselI, Log, and Exp are functions used in close form expressions. Similarly, substituting s for ak[z,q], we obtained the number of operations in the accelerated algorithm.

## 3. Applications of the Accelerating Procedure and Operation Reduction with Microsimulation Semi-Symbolic Analysis

In this section, the operation reduction using fast computation of an iteration-based simulation method with microsimulation-semi-symbolic analysis was applied to two processing problems to illustrate the shorter computation time of the algorithm, and to demonstrate the variety of applications for which the operation may be used. A case with complex calculation is illustrated in the example with non-coherent Amplitude-Shift Keying (ASK) with shadowing, interference, and correlated noise. The second example treats second-order statistics in the SC macrodiversity system operating over Gamma shadowed Nakagami-m fading channels [20].

### 3.1. Non-Coherent Amplitude Shift Keying (ASK) with Shadowing, Interference, and Correlated Noise

**Figure 3:**
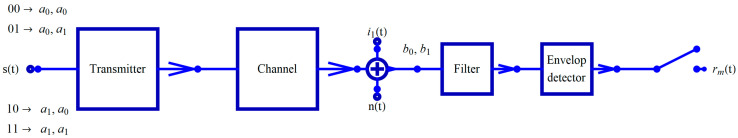
Non-coherent amplitude shift keying (ASK) system with interference *i*_1_(*t*).

Non-coherent ASK is a modulation scheme used to send digital information between digital equipment and it is shown on Figure 3. Similar part of the system, where real-time estimation is needed, can be found in [21]. The data is transmitted by the non-coherent system without a carrier in a binary manner.

Shadowing with interference is one of the most common models used in wireless communications to describe the phenomenon of multiple scattering [21,22,23,24]. The basic components of the system are shown in Figure 1. Both shadowing and interference cause strong fluctuations in the amplitude of the useful signal. This occurs in urban areas and is described as a log-normal distribution. In our analysis, we performed an outage probability. Transmitting signals using two symbols were observed in the non-coherent ASK system [25,26]. The noise, as a narrow-band stochastic process, is correlated and the coefficient of correlation is denoted by *R* (*R* ≠ 1). Mathematically, the noise can be described as *n_i_*(*t*) = *x_i_*(*t*)·cos(*ωt*) − *y_i_*(*t*)·sin(*ωt*). The receiver is sheltered, and no optical visibility exists toward the transmitter, but interference *i*_1_(*t*) = *A*_1_·cos(*ωt*) is present. If the system sends logical zero, then the signal *s*_0_(*t*) = *a*_0_·cos(*ωt*) has been sent, but if the system sends a logical unit, then the signal *s*_1_(*t*) = *a*_1_·cos(*ωt*) has been sent. The parameters *a*_0_ and *a*_1_ are the signal elements from which the code words are formed. The receiver detects information signal *b*_0_·cos(*ωt*) and *b*_1_·cos(*ωt*) with envelops *z*_0_ and *z*_1_ after passing through a transmitting channel. The *b_m_* (*m* = 0, 1) are the elements of the detected signals. The receiver system includes a filter and detector envelope. In the receiver input, the signal is:

*r_m_*(*t*) = *b_m_*·cos(*ωt*) + *A*_1_·cos(*ωt*) + *x_m_*·cos(*ωt*) − *y_m_*·sin(*ωt*) = *z_m_*·cos(*ωt* + *φ*_m_), *m* = 0, 1 (3)

with envelopes *z*_0_ and *z*_1_, and phases *φ*_0_ and *φ*_1_, respectively.

The general form of the condition joint probability density function is:

(4) $$p(x_{0},x_{1},y_{0},y_{1})=\frac{1}{4\pi^{2}\sigma^{4}\sqrt{1-R^{2}}}\exp\left\{-\frac{x_{0}^{2}+x_{1}^{2}+y_{0}^{2}+y_{1}^{2}-2R(x_{0}x_{1}+y_{0}y_{1})}{2\sigma^{2}(1-R^{2})}\right\}$$

where *R* is the coefficient of correlation and *σ* is variance. To ensure the set of expressions is solved continuously, using the polar coordinates is necessary, as follows:

(5) $$\begin{array}{l} x_{0}=z_{0}\cos\varphi_{0}-b_{0}-A_{1}\\ y_{0}=-z_{0}\sin\varphi_{0}\\ x_{1}=z_{1}\cos\varphi_{1}-b_{1}-A_{1}\\ y_{1}=-z_{1}\sin\varphi_{1} \end{array}$$

The next step was determining the condition joint probability density function (JPDF). Substituting Equation (5) into Equation (4), we obtained:

*p*(*r*_0_, *r*_1_/*b*_0_, *b*_1_, *φ*_0_, *φ*_1_, *A*_1_) = *p*(*x*_0_, *y*_0_, *x*_1_, *y*_1_)·|*J*| (6)

where |*J*| is Jacobian. A joint probability density function has a log-normal distribution described as [22]:

(7) $$\begin{array}{l} p_{ij}(b_{0},b_{1}/A_{1})=\frac{1}{2\pi \sigma^{2}(b_{0}+A_{1})(b_{1}+A_{1})\sqrt{1-R^{2}}}\times \\ \;\times \exp\left\{-\frac{{\left(10\log(b_{0}+A_{1})-a_{i}\right)}^{2}+{\left(10\log(b_{1}+A_{1})-a_{j}\right)}^{2}}{2\sigma^{2}\left(1-R^{2}\right)}\right\}\\ \;\times \exp\left\{\frac{2R\left(10\log(b_{0}+A_{1})-a_{i}\right)\left(10\log(b_{1}+A_{1})-a_{j}\right)}{2\sigma^{2}\left(1-R^{2}\right)}\right\} \end{array}$$

For *i* = *j* = 0, the code word 00 was sent; for *i* = 1 and *j* = 0, the code word 01 was sent; for *i* = 0 and *j* = 1, the code word 10 was sent; and for *i* = 1 and *j* = 1, the code word 11 was sent. So:

(8) $$\begin{array}{l} p(z_{0},z_{1}/b_{0},b_{1},\varphi_{0},\varphi_{1},A_{1})=\frac{z_{0}z_{1}}{{\left(2\pi \right)}^{2}\sigma^{4}\sqrt{1-R^{2}}}\times \\ \;\times \exp\left\{-\frac{z_{0}{}^{2}+z_{1}{}^{2}-2R(b_{0}+A_{1})(b_{1}+A_{1})-2Rz_{0}z_{1}\cos(\varphi_{0}-\varphi_{1})}{2\sigma^{2}\left(1-R^{2}\right)}\right\}\times \\ \;\times \exp\left\{\frac{2(1+R)(b_{0}+b_{1}+2A_{1})(z_{0}\cos\varphi_{0}+z_{1}\cos\varphi_{1})}{2\sigma^{2}\left(1-R^{2}\right)}\right\} \end{array}$$

The last expression can be transformed using a modified Bessel function [27] before derivation of the closed form expression:

(9) $$e^{x\cos\alpha }=\sum_{n=-\infty }^{\infty }I_{n}(x)\cos n\alpha$$

and applying trigonometric transformation:

(10) $$\cos\alpha \cos\beta \cos\gamma =\frac{1}{4}[\cos(\alpha +\beta +\gamma )+\cos(\alpha -\beta +\gamma )+\cos(\alpha +\beta -\gamma )+\cos(\alpha -\beta -\gamma )]$$

Equation (8) becomes:

(11) $$\begin{array}{l} p(z_{0},z_{1}/b_{0},b_{1},\varphi_{0},\varphi_{1},A_{1})=\\ \;=C\cdot e^{C_{0}}\sum_{n=-\infty }^{\infty }\sum_{m=-\infty }^{\infty }\sum_{k=-\infty }^{\infty }I_{n}(C_{1})I_{m}(C_{2})I_{k}(C_{3})\times \cos[n\varphi_{0}]\cos[m\varphi_{1}]\cos[k(\varphi_{0}-\varphi_{1})] \end{array}$$

where:

(12) $$\begin{array}{l} C=\frac{z_{0}z_{1}}{{\left(2\pi \right)}^{2}\sigma^{4}\sqrt{1-R^{2}}}\\ C_{0}=-\frac{z_{0}{}^{2}+z_{1}{}^{2}-2R(b_{0}+A_{1})(b_{1}+A_{1})}{2\sigma^{2}(1-R^{2})}\\ C_{1}=\frac{\left(b_{0}+A_{1})(b_{1}+A_{1}\right)}{\sigma^{2}(1-R)}z_{0}\\ C_{2}=\frac{\left(b_{0}+A_{1})(b_{1}+A_{1}\right)}{\sigma^{2}(1-R)}z_{1}\\ C_{3}=\frac{R\cdot z_{0}z_{1}}{\sigma^{2}(1-R^{2})} \end{array}$$

Using the Bessel identity *I_n_*(*x*) = *I*_-*n*_(*x*), it follows that:

(13) $$p(z_{0},z_{1}/b_{0},b_{1},A_{1})=2\pi^{2}C\cdot e^{C_{0}(b_{0},b_{1},A_{1})}\times \sum_{n=0}^{\infty }I_{n}\left[C_{1}(b_{0},b_{1},A_{1})\right]\cdot I_{n}\left[C_{2}(b_{0},b_{1},A_{1})\right]\cdot I_{n}\left[C_{3}\right]$$

The present interference is described with the Rayleigh distribution over the probability density function (PDF) [23,24] as:

(14) $$p(A_{1})=\frac{A_{1}}{\sigma^{2}}\exp\left\{-\frac{A_{1}^{2}}{2\sigma^{2}}\right\};\;0\leq A_{1}\leq \infty$$

To eliminate the interference, performing averaging is necessary for all values of interference *A*_1_.

(15) $$p_{ij}(z_{0},z_{1}/b_{0},b_{1})=\int_{0}^{\infty }p(z_{0},z_{1}/b_{0},b_{1},A_{1})\cdot p(A_{1})dA_{1}$$

The integral in Equation (15) is solved using integral:

(16) $$\int_{-\pi }^{\pi }\int_{-\pi }^{\pi }\cos n\varphi_{0}\cos m\varphi_{1}\cos k(\varphi_{0}-\varphi_{1})d\varphi_{0}d\varphi_{1}=\left\{ \begin{array}{l} \pi^{2},\;\left|n\right|=\left|m\right|=\left|k\right|\\ 0,\;n\neq m\neq k \end{array}\right.$$

The distribution is obtained by averaging *φ*_0_ and *φ*_1_ for all values between −*π* and *π*.

(17) $$p(z_{0},z_{1}/b_{0},b_{1})=\int_{-\pi }^{\pi }\int_{-\pi }^{\pi }p(z_{0},z_{1}/b_{0},b_{1},\varphi_{0},\varphi_{1})d\varphi_{0}d\varphi_{1}$$

For all code word combinations, distributions of envelopes are obtained by integrating all values between *b*_0_ and *b*_1_. So, when the code word |*ij*| (*i* = 0, 1; *j* = 0, 1) has been sent, marked with *H_i_H_j_* in Equation (18), and when the same is detected in the input of the receiver marked with *D_i_D_j_*, the detection of the signals is described as:

(18) $$P(D_{i}D_{j}/H_{i}H_{j})=p_{ij}(z_{0},z_{1})=\int_{0}^{\infty }\int_{0}^{\infty }p(z_{0},z_{1}/b_{0},b_{1})\cdot p_{ij}(b_{0},b_{1})db_{0}db_{1}$$

The outage probability is:

(19) $$P_{outage}=1-\sum_{i=0}^{1}\sum_{j=0}^{1}P(H_{i}H_{j})P(D_{i}D_{j}/H_{i}H_{j})$$

where $P(H_{i}H_{j})=P(H_{i})\cdot P(H_{j})=\frac{1}{2}\cdot \frac{1}{2}=\frac{1}{4}$, *i* = 0, 1, and *j* = 0, 1. For the outage probability, Equation (19) represents closed form expression and is often not present in the closed form solution. Closed form expression represents an implicit solution that is contained in a mathematical expression [12]. A closed form solution provides a solved problem in terms of functions and mathematical operations from a given and generally-accepted set [28]. In other words, a closed form solution provides an explicit solution to an observed problem, whereas closed form expression shows an implicit or insufficient solution.

**Figure 4:**
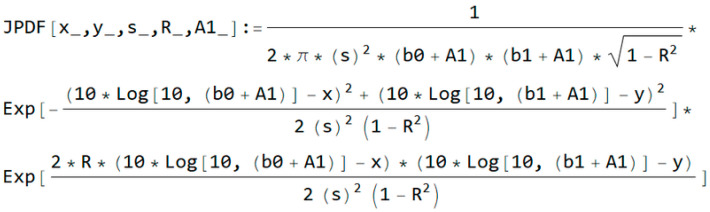
Condition joint probability density function using Wolfram language for shadowing and interference.

From Equation (7), the joint probability density function is shown in Figure 4.

**Figure 5:**
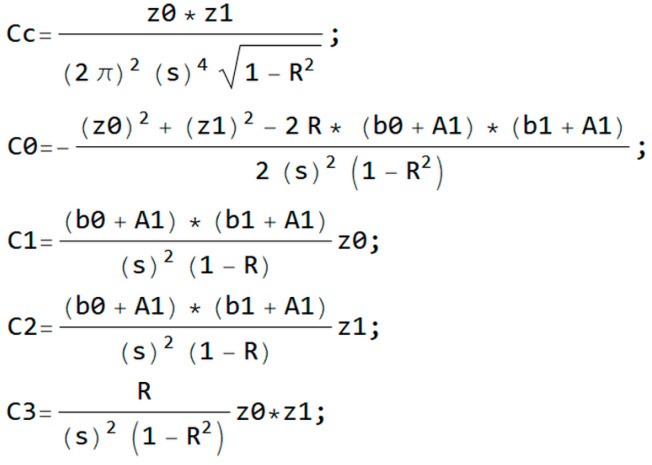
Changing of coefficients for simplification.

Figure 5 shows the manipulating of Equation (8) and substituting into Equation (12) for the changing of coefficients for simplification.

**Figure 6:**
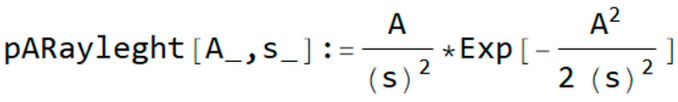
Rayleigh distribution for interference coded by Wolfram language.

**Figure 7:**
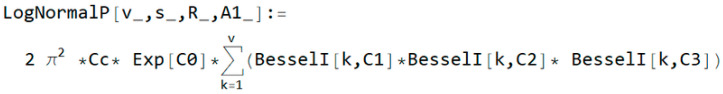
Log-normal distribution for non-coherent ASK in the presence of shadowing and interference.

**Figure 8:**
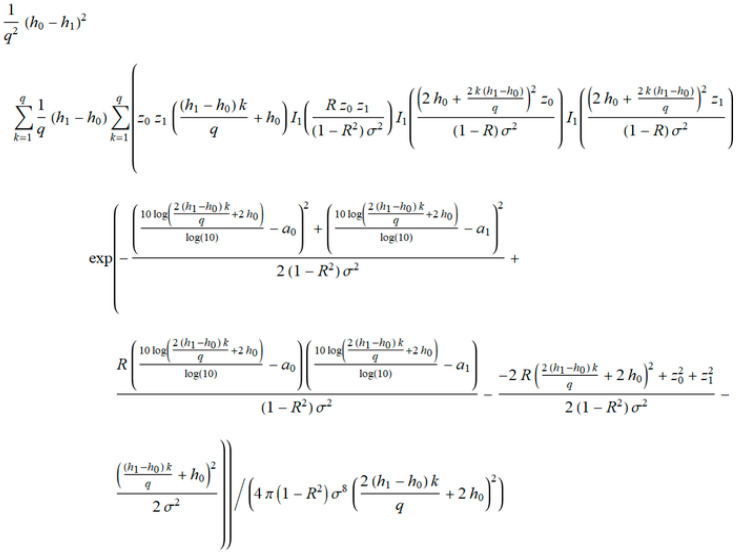
Closed form solution of probability density function (*PDF_outage_*) of a non-coherent ASK system.

Interference *A*_1_ is present per Equation (14) and described by Wolfram language code in Figure 6,

Averaging all *A*_1_ values is necessary, according to Equation (15). The general form of the condition joint probability density function is defined in Equation (14), and is described in Figure 7.

s is marked variance σ, R is the correlation coefficient, and v is the order of the iterations. The finalization of IBSM obtains closed form expressions of the probability density function, and outage probability in term of iterations (Figure 8).

The closed form of *PDF_outage_* in Figure 8 provides the next parameters: iteration *q*, *h*_0_, and *h*_1_ are the resolution of the iteration, *z*_0_ and *z*_1_ are envelopes, *R* is the coefficient of correlation, and *σ* is variance. This expression cannot be manually obtained by using numerical tools. The resultant closed form solution of *P_outage_* is an expression that is ready for further processing. Accordingly, the viewpoint is an insight into the parameters and variables that participate in obtaining all the features of this case study. Drawing the characteristics is now possible, but this calculation would take too long, regardless of the chosen accuracy. On the other hand, for greater accuracy, a number of iterations is required, which is not beneficial for this form of expression.

**Figure 9:**
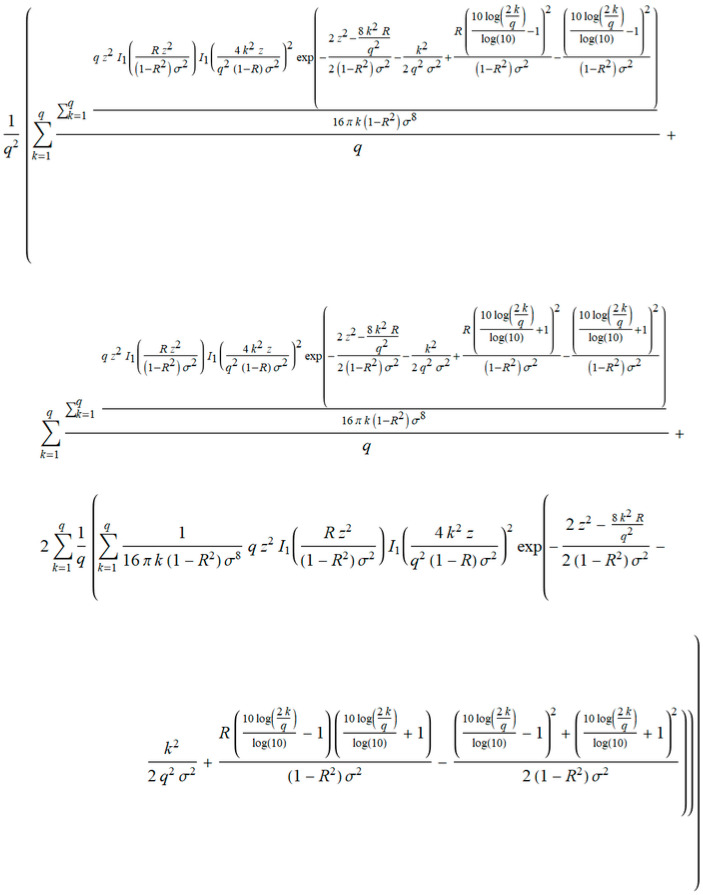
Closed form solution of outage probability *P_outage_* of a non-coherent ASK system with shadowing and interference.

Finally, the closed form solution of *P_outage_* is shown in Figure 9.

In our case, a member *a_k_* represents a general member of the series in *P_outage_*, from the closed form solution in Figure 9.

Convergence testing of the *a_k_* verified that:

(20) $$\underset{\begin{array}{l} k\to q\\ q\to \infty \end{array}}{\lim}a_{k}=0$$

Convergence testing was performed with assumptions that 0 ≤ *R* < 1, *σ* > 0, *z* ≥ 0, and *q* ≥ 1.

The selection of the auxiliary function is one of the most important aspects of the MSSA [29]. In testing many series, the authors of this paper highlighted the series that shows the best performance to accelerate convergence, meaning a shorter computation time with the optimum number of iteration. Comparative analysis of different auxiliary series can be the subject of particular surveys, and the reader(s) are encouraged to do so. Therefore, in our case, the auxiliary series is:

(21) $$C=\sum_{k=1}^{\infty }\frac{1}{k^{5}2^{k-1}}$$

**Figure 10:**
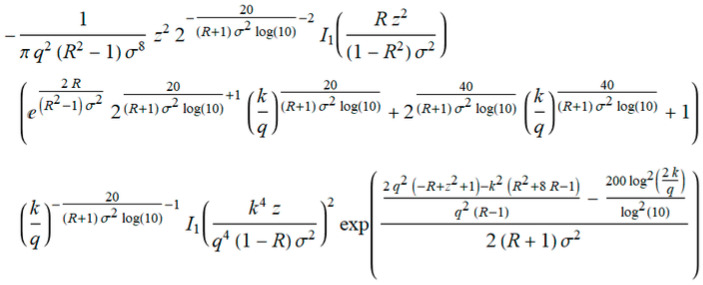
General term in a series of *P_outage_* marked as *a_k_*.

**Figure 11:**
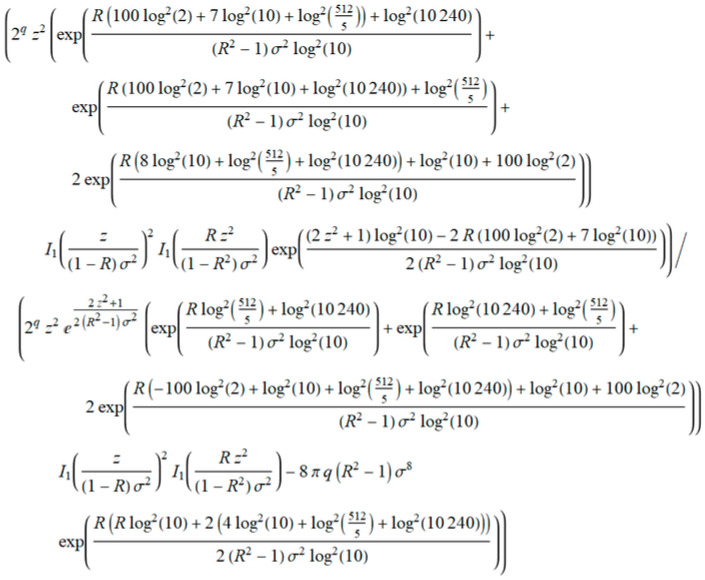
The element Kummer’s transformation *ρ*.

The series converges to 2log2. To fully use Equation (2), we made a minor modification to the member *b_k_*, with respect to the convergence theorems that have been mentioned above. The new member becomes *b_k_* → *a_k_* + *c_k_*, so:

(22) $$s=\sum_{k=0}^{\infty }a_{k}=\rho \sum_{k=0}^{\infty }a_{k}+\sum_{k=0}^{\infty }\left(1-\rho \frac{a_{k}+c_{k}}{a_{k}}\right)a_{k}=\rho C+\sum_{k=0}^{\infty }\left(1-\rho \frac{a_{k}+c_{k}}{a_{k}}\right)a_{k}$$

where *c_k_* is general term in Equation (21). We obtain the general member of *P_outage_* marked as *a_k_* in Figure 10, separating it from Figure 9. Following the next step in MSSA, we derived the term *ρ* (Figure 11).

We checked that the value *ρ* tends to 1 after convergence testing. The quicker computation was performed by assuming how much iteration is required to calculate the outage probability *P_outage_* obtained by the IBSM. Otherwise, a large number of iterations are required to calculate the closest exact values of *P_outage_*, but the computation is time consuming. Then, the resulting *P_outage_* equalizes with a new series obtained by the Kummer's transformation, and performs point matching for the various values of the envelopes, followed by a new reduced number of iterations. After that, the verification of the obtained results was performed by checking the relative error, which determined the degree of adjustability of the algorithm [29]. Finally, we checked the number of operations of calculations in the expression in Figure 9, and then obtained a reduced number of operations with a new decreased number of iterations.

After all symbolic derivations, we used closed form solutions to directly obtain results in the first attempt. To obtain concrete numerical results, we needed to set the initial parameters. We supposed that the closest exact value was obtained after 500 iterations by using the outage probability *P_outage_* in Figure 9, and the resolution of the iteration was *h*_0_ = 0 and *h*_1_ = 1. We also used *z*_0_ = *z*_1_ = *z* to simplify the analysis. The next step was calculating the new numbers of iterations that are reduced for various values of the envelope *z*. This was performed using the command FindRoot[s==Poutage,{q,1}]. s is a new expression obtained by Kummer’s transformation in Equation (22), and *P_outage_* is a closed form solution in Figure 8. We took the range of values *z* = {1, 15} for a concrete case [29]. Experiments were performed for various values of the coefficient of correlation *R* (*R* = 7/10,8/10) and the variance *σ* (*σ* = 2, 3). All calculations were performed with a precision of 10^−6^. All tests were performed on a PC with: Intel^®^ Core™ i5-6500 CPU@ 3.2 GHz, 8 GB RAM, 64-bit Operating System, Windows 10, and Mathematica Wolfram 11.1. The reduced number of iterations are shown in Table 1.

**Figure 12:**
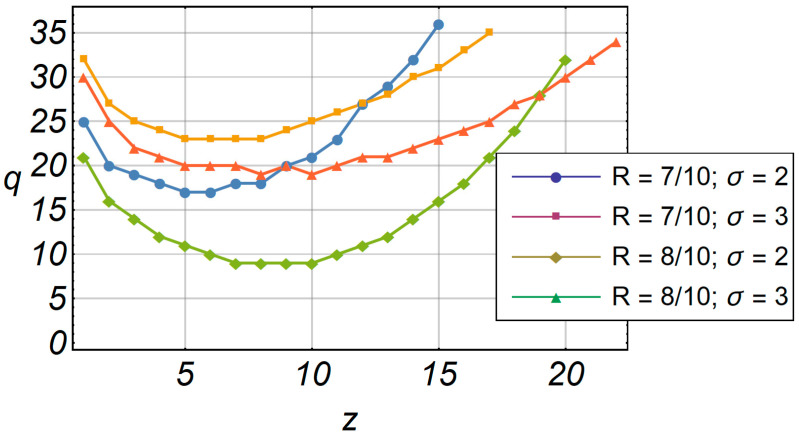
The number of iterations in term of envelope *z*.

In Figure 12, the changing iteration values in terms of the envelope *z* are shown for the accelerated algorithm. Notably, the reduced number of iterations is not the same for each envelope value. The minimum number of iterations is *z* = 10, where the value provides a true detection. However, the number of iterations is in range of 9 to 35 if we observe the total range of the envelope, which is a significant reduction compared to the original 500 iterations.

Since the absolute error is not precisely characterized by accuracy, the relative error is used as:

(23) $$\delta =\frac{s-P_{outage}}{P_{outage}}$$

**Figure 13:**
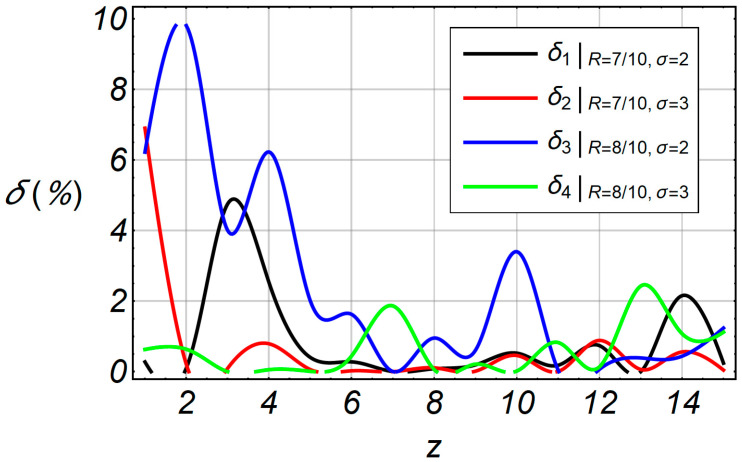
Relative error functions in term of the envelope *z*.

**Figure 14:**
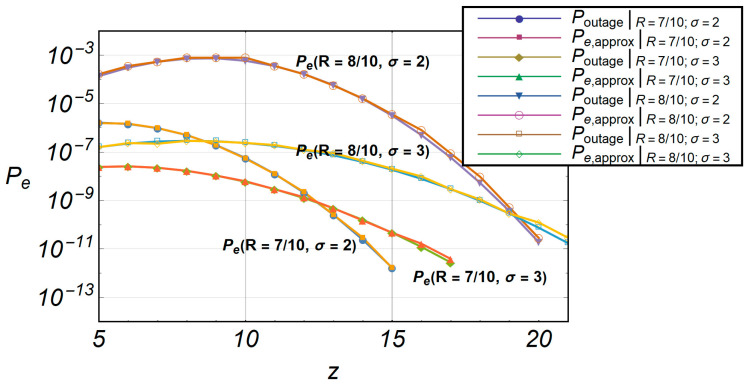
Comparative characteristics of *P_outage_* and accelerated outage probability *s*.

Relative errors do not exceed more than 10% of the value as it is shown in Figure 13. This indicates that the algorithm is quite accurate. In Figure 14, the comparative characteristics of *P_outage_* and *s* are shown. The accelerated algorithm *s* is marked as *P_e,approx_*.

The total calculation of formula *P_outage_* required 1193.97 s, or 19 min and 54 s, so the average time per iteration was 70.2335 s. The sped up algorithm’s total calculation time for the accelerated formula was 1.25 s, so the average time per iteration was 0.0735294 s. Wolfram language code for time consumed is: Table[Timing[N[Poutage]],{z,15}] // Total. Command Table provides a calculation for any value of envelope z, and command Timing provides the exact time of calculation. Command Total summarizes total time per envelope. Similarly, changing the parameter Poutage with s for the accelerated algorithm in the previous WL command line provides the time consumed for fast computation. Our algorithm is accelerated as:

(24) $$Ratio=\frac{time(P_{outage})}{time(s)}=\frac{1193.97}{1.25}\approx 955times$$

**Figure 15:**
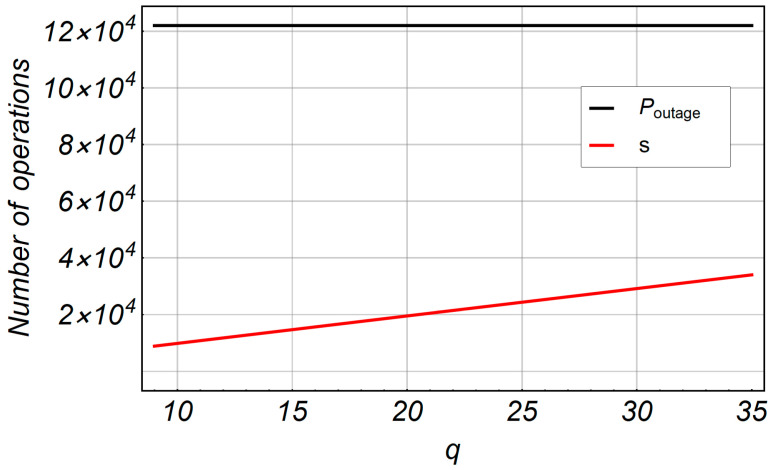
Number of operations in terms of number of iterations *q* for fast computation. The number of iterations is fixed with *q* = 500 for *P_outage_*.

Figure 15 shows the number of operations in terms of the number of iterations *q* for fast computation. The number of iterations is fixed at *q* = 500 for *P_outage_* because we initially assumed that this number of iterations was satisfied for the closest exact value of *P_outage_*. The number of operations for fast computation of IBSM is less than *P_outage_*. For 500 iterations, we counted 120,000 math operations for *P_outage_*. The number of math operations changes in the range of 9000 to 34,000, which is the result of variety in the number of iterations for fast computation.

### 3.2. Second-Order Statistics in Wireless Channels

The level crossing rate (LCR) and the average duration of fade (ADF) are important second-order statistical characteristics describing the fading channel in mobile communications. These values are suitable for designing mobile radio communication systems and for analyzing their performance. In digital telecommunications, a sudden drop in the value of the received signal directly leads to a drastic increase in the probability of error. For optimizing the coding system required to correct errors, the number of times the received signal passes through the given level in time and how long, on average, the signal is below the specified level must be known. The LCR and ADF are the appropriate measures closely related to the quality of the received signal [24].

The LCR of signal *Z*(*t*), marked as *N*_Z_(*z*), is defined as the signal speed crossing through level *z* with a positive derivative at the intersection point *z*. The ADF, marked as *T*_Z_(*z*), represents the mean time for which the signal overlay is below the specified *z* level.

The LCR at envelope *z* is mathematically defined by [22]:

(25) $$N_{Z}(z)=\int_{0}^{\infty }\overset{•}{z}p_{z\overset{•}{Z}}(z,\overset{•}{z})d\overset{•}{z}$$

where *z* is the envelope of the received signal, $\overset{•}{z}$ is its derivative in time, and $p_{z\overset{•}{Z}}(z,\overset{•}{z})$ is the joined probability density function. The average fade duration (AFD) is determined as [22]:

(26) $$T_{Z}(z)=\frac{F_{Z}(z\leq Z)}{N_{Z}(z)}$$

where *F_Z_*(*z* ≤ *Z*) represents the probability that the signal level *Z*(*t*) is less than the level *z*. Evaluation and calculation of LCR and ADF are trivial in an environment where no large reflections exists with a large number of transmission channels and shadowing, which simplifies the mathematical description of the distribution of the signal. However, in complex environments, obtaining LCR and ADF characteristics is time-consuming. An example of a complex environment is described in Stefanovic et al. [20]. In this example, the LCR and ADF expressions were obtained. Their analytical shapes are closed forms, but the complexity shows a long computation time. Thus, the LCR value is normalized by the Doppler shift frequency *f_d_* [20] through Equation (15):

(27) $$\begin{array}{l} \frac{N_{Z}(z)}{f_{d}}=\frac{z^{M_{1}-1}}{\Gamma (M_{1})\Gamma (c_{1})\Gamma (c_{2})}{\left(\frac{N_{1}m_{1}}{r_{1}}\right)}^{M_{1}-1}\sqrt{\frac{2\pi }{m_{1}}}\times \\ \times \sum_{k=0}^{\infty }\frac{{\left(N_{1}m_{1}z/r_{1}((1/\Omega_{01})+(1/\Omega_{02}))\right)}^{\frac{M_{1}-c_{1}-c_{2}+k-1/2}{2}}}{c_{2}{\left(1+c_{2}\right)}_{k}\Omega_{01}^{c_{1}}\Omega_{02}^{k+c_{2}}}K_{\left(M_{1}+c_{1}+c_{2}+k-1/2\right)}\left(2\sqrt{\frac{N_{1}m_{1}z(\Omega_{01}+\Omega_{02})}{r_{1}\Omega_{01}\Omega_{02}}}\right)+\\ +\frac{z^{M_{2}-1}}{\Gamma (M_{2})\Gamma (c_{1})\Gamma (c_{2})}{\left(\frac{N_{2}m_{2}}{r_{2}}\right)}^{M_{2}}\sqrt{\frac{2\pi }{m_{2}}}\times \\ \times \sum_{k=0}^{\infty }\frac{{\left(N_{2}m_{2}z/r_{2}((1/\Omega_{01})+(1/\Omega_{02}))\right)}^{\frac{M_{2}-c_{1}-c_{2}+k-1/2}{2}}}{c_{2}{\left(1+c_{2}\right)}_{k}\Omega_{01}^{c_{1}}\Omega_{02}^{k+c_{2}}}K_{\left(M_{2}+c_{1}+c_{2}+k-1/2\right)}\left(2\sqrt{\frac{N_{2}m_{2}z(\Omega_{01}+\Omega_{02})}{r_{2}\Omega_{01}\Omega_{02}}}\right) \end{array}$$

where Γ(*x*) denotes the Gamma function, *M_i_* is $(m_{i}N_{i}^{2})/r_{i}$, *m_i_* is the Nakagami-m fading severity parameter, *N_i_* denotes the number of identically assumed channels at each microlevel, *r_i_* is related to the exponential correlation *ρ_i_*, *c_i_* denotes the order of Gamma distribution, Ώ_0*i*_ is related to the average powers of the Gamma long-term fading distributions, and *K_v_*(*x*) is the modified Bessel function of the second order. Similarly, the AFD is obtained as [20] per Equation (16):

(28) $$\begin{array}{l} T_{Z}(z)=\frac{F_{Z}(z\leq Z)}{N_{Z}(z)}\\ P_{z}(Z)=\frac{2{\left(N_{1}m_{1}/r_{1}\right)}^{M_{1}}}{\Gamma (M_{1})\Gamma (c_{1})\Gamma (c_{2})M_{1}}\times \\ \sum_{k=0}^{\infty }\sum_{l=0}^{\infty }{\left(\frac{N_{1}m_{1}}{r_{1}}\right)}^{k}\frac{{\left(N_{1}m_{1}z/r_{1}((1/\Omega_{01})+(1/\Omega_{02}))\right)}^{\frac{c_{1}+c_{2}++l-k-M_{1}}{2}}}{c_{2}{\left(1+c_{2}\right)}_{k}\Omega_{01}^{c_{1}}\Omega_{02}^{l+c_{2}}}K_{\left(c_{1}+c_{2}+l-k-M_{1}\right)}\left(2\sqrt{\frac{N_{1}m_{1}z(\Omega_{01}+\Omega_{02})}{r_{1}\Omega_{01}\Omega_{02}}}\right)+\\ \frac{2{\left(N_{2}m_{2}/r_{2}\right)}^{M_{2}}}{\Gamma (M_{2})\Gamma (c_{1})\Gamma (c_{2})M_{2}}\times \\ \sum_{k=0}^{\infty }\sum_{l=0}^{\infty }{\left(\frac{N_{2}m_{2}}{r_{2}}\right)}^{k}\frac{{\left(N_{1}m_{1}z/r_{1}((1/\Omega_{01})+(1/\Omega_{02}))\right)}^{\frac{c_{1}+c_{2}+l-k-M_{2}}{2}}}{c_{2}{\left(1+c_{2}\right)}_{k}\Omega_{01}^{c_{1}}\Omega_{02}^{l+c_{2}}}K_{\left(c_{1}+c_{2}+l-k-M_{2}\right)}\left(2\sqrt{\frac{N_{1}m_{1}z(\Omega_{01}+\Omega_{02})}{r_{1}\Omega_{01}\Omega_{02}}}\right)+ \end{array}$$

**Figure 16:**
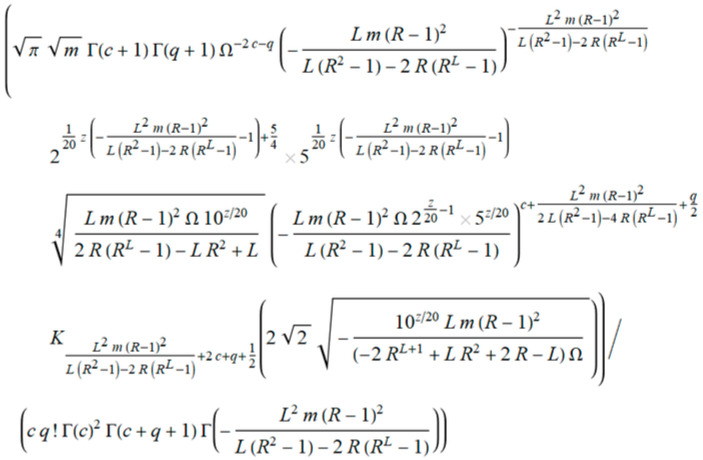
General term of the level crossing rate (LCR) marked as *a_k_*.

As in the previous example, we defined a general term *a_k_* from Equation (27), shown in Figure 16.

**Figure 17:**
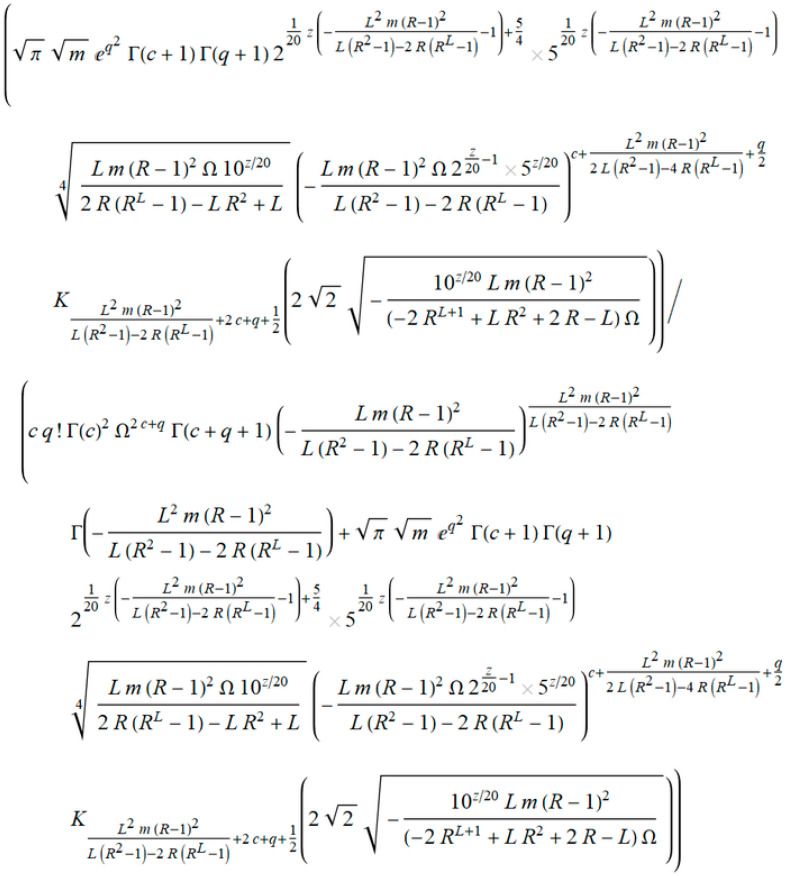
The element Kummer’s transformation *ρ*.

Using the expression in Figure 17, we derived the term *ρ* that tends to 1 when *q* → ∞.

In this case, Equations (27) and (28) have already been provided in advance in a closed form where the iteration parameter *q* is present, so applying the IBSM would be excessive. To compute the closest exact values of LCR and AFD, 100 iterations were required in Stefanovic et al. [20]. Using Kummer’s transformation, both LCR and AFD were calculated in the first iteration. All computations were performed using the values of *m* = 1, *L* = 2, Ώ = 1, *c* = 2, and *R* = 1/5. An auxiliary series was used:

(29) $$C=\sum_{k=1}^{\infty }e^{-k^{2}}$$

The series *C* converges to (1/2)·(ϑ_3_(0, e^−1^)–1), where ϑ*_a_*(*u*, *x*), (*a* = 1,…,4) is the theta function, defined as [30]:

(30) $$\vartheta_{3}(u,x)=1+2\sum_{k=1}^{\infty }x^{k^{2}}\cos(2k\cdot u)$$

**Figure 18:**
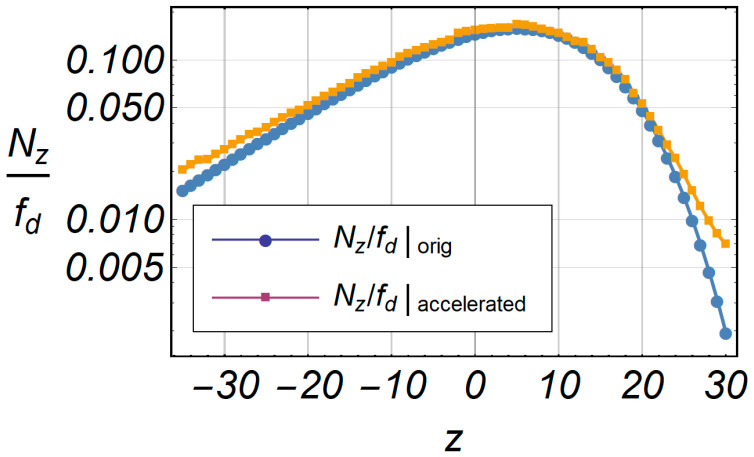
Comparative characteristics of LCR and accelerated LCR.

**Figure 19:**
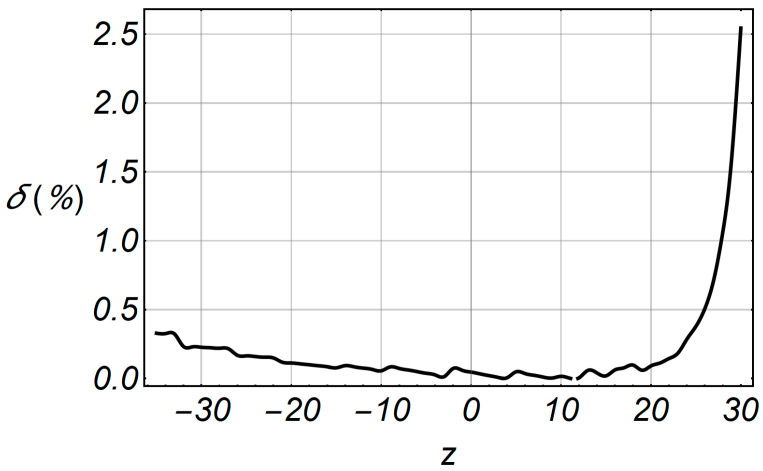
Relative error function of LCR in term of the envelope *z*.

Figure 18 shows the comparative characteristics of LCR and accelerated LCR. The deviation of the accelerated series is small in relation to the original series, and the relative error is shown in Figure 19, in the specified range of envelope –35 ≤ *z* ≤ 30.

The total calculation of the LCR formula required 30.6563 s, so the average time per iteration was 0.437946 s. The total calculation time with the sped up algorithm in the accelerated formula was 1.53125 s, so the average time per iteration was 0.021875 s. Our algorithm is accelerated as:

(31) $$Ratio=\frac{time(LCR_{orig})}{time(LCR_{accelerated})}=\frac{30.6563}{1.53125}\approx 20times$$

**Figure 20:**
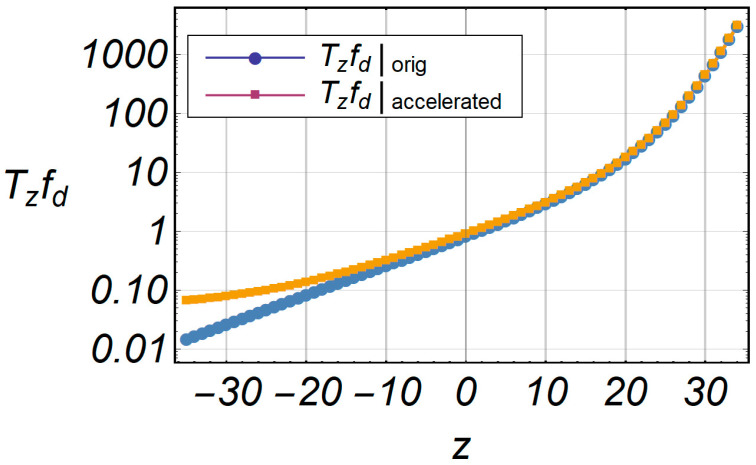
Comparative characteristics of the average fade duration (AFD) and accelerated AFD.

**Figure 21:**
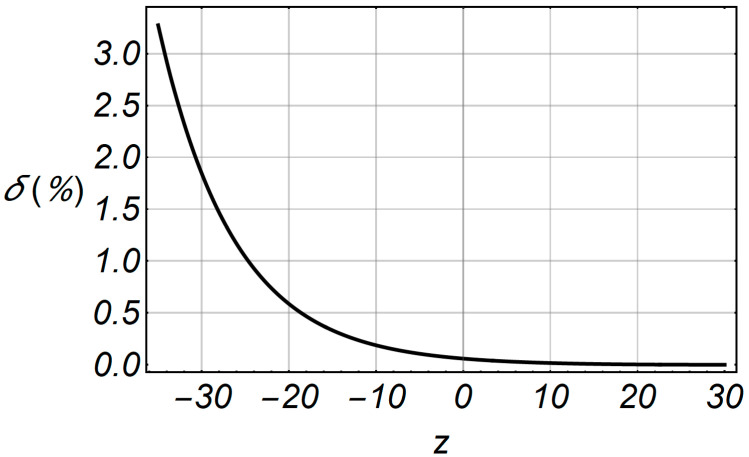
Relative error function of AFD in terms of envelope *z.*

Figure 18 shows the number of operations of LCR (*N_Z_*) in terms of the number of iterations *q* for fast computation. The number of iterations was fixed at *q* = 100 for *LCR_orig_* because we initially assumed that this number of iterations satisfied the closest exact value of *LCR_orig_*. For 100 iterations, we counted 20,200 math operations for *LCR_orig_*. The number of math operations was 1184 for *LCR_accelerated_* calculated in the first iteration using fast computation. Using the same method, the AFD was obtained by applying Equation (22). Figure 20 shows the comparative characteristics of AFD and accelerated AFD. A small deviation in the range of −35 ≤ *z* ≤ −28 was observed, perceived through the relative error in Figure 21.

The total calculation of formula *AFD_orig_* required 19,553.1 s, or 5 h and 25 min, so, the average time per iteration was 279.33 s, or 4 min and 19.33 s. The sped up algorithm total calculation time with the accelerated formula was 1.29688 s, so, the average time per iteration was 0.0185268 s. An obvious difference in the time calculation exists because the number of sums for AFD increased in Equation (27), where we have sums for *k*, *l*, and *q*. In this case, our algorithm is accelerated as:

(32) $$Ratio=\frac{time(AFD_{orig})}{time(AFD_{accelerated})}=\frac{19,553.1}{1.29688}\approx 15\cdot 10^{3}times$$

For 100 iterations, we counted the 344 × 10^6^ math operations for *AFD_orig_*. The number of math operations was 5619 for *LCR_accelerated_* calculated in the first iteration for fast computation.

## 4. Conclusions

This paper presents a new method to accelerate the computation and reduce the number of calculation operations in the iteration-based simulation method. The method was developed to simulate the systems and processes when obtaining mathematical formulas in the final closed form is not possible. Often, many phenomena show that closed form expressions and simulations are executed with numerical based tools. In these cases, the users do not have insight into the phenomena that affect the flow of processes, which can lead to incorrect assumptions and results. The method provides insight into processes and systems using symbolic processing, with significant acceleration and reduction in the number of computation operations required. For symbolic derivation, the computer algebra system was used, and Kummer’s transformation was used to shorten the computation time. The complete method to reduce the number of operations and shorten the computation time was illustrated in two examples. Both cases require complex and time-consuming calculations. Due to the large number of operations, the memory resources can also play a significant role in the speed of the calculation. The acceleration of the algorithm and the reduction of the number of operations significantly affected efficiency in terms of time savings and the rapid production of results. The method can be used in many fields where fast computation in one-step simulation runs is required.

## Figures and Tables

**Table 1:** Reduced number of iterations.

*z*	*q*_1_ *R* = 7/10; σ = 2	*q*_2_ *R* = 7/10; σ = 3	*q*_3_ *R* = 8/10; σ = 2	*q*_4_ *R*= 8/10; σ = 3
1	25	32	21	30
2	20	27	16	25
3	19	25	14	22
4	18	24	12	21
5	17	23	11	20
6	17	23	10	20
7	18	23	9	19
8	18	23	9	19
9	20	24	9	20
10	21	25	9	19
11	23	26	10	20
12	27	27	11	21
13	29	28	12	21
14	32	30	14	22
15	36	31	16	23
